# Adenovirus viremia may predict adenovirus pneumonia severity in immunocompetent children

**DOI:** 10.1186/s12879-021-05903-4

**Published:** 2021-02-25

**Authors:** Ruimu Zhang, Hongmei Wang, Shufeng Tian, Jikui Deng

**Affiliations:** Department of Infectious Diseases, Shenzhen Children’s Hospital, Shenzhen, China

**Keywords:** Adenovirus, Viremia, Pneumonia, Immunocompetent children

## Abstract

**Background:**

Previous studies have demonstrated an association between adenovirus viremia and disease severity in immunocompromised children. However, few studies have focused on this association in immunocompetent children. This study explored the association between adenovirus viremia and adenovirus pneumonia severity in immunocompetent children.

**Methods:**

We performed a retrospective, observational study of immunocompetent children with adenovirus pneumonia admitted to Shenzhen Children’s Hospital in Shenzhen, China. Pneumonia was classified as severe or mild based on the Chinese guideline for the classification of pneumonia severity. Serum samples from all the children included in the study were tested for adenovirus DNA with a quantitative polymerase chain reaction. Clinical manifestations, laboratory examinations, and disease severity were compared between children with severe and mild pneumonia.

**Results:**

A total of 111 immunocompetent children with adenovirus pneumonia (60 severe, 51 mild) were included. The median age was 40 months, and 64 patients were male. Five patients were admitted to the intensive care unit, and two underwent endotracheal intubation. All patients were discharged after recovery or improvement. Univariate analysis and binary logistic regression analysis showed that leukocytosis (OR = 1.1; 95% CI: 1.0 to 1.2; *P* = 0.033), co-infection with *Mycoplasma pneumoniae* (OR = 5.0; 95% CI: 2.1 to 12.3; *P* <  0.001), and high blood viral load (OR = 1.5; 95% CI: 1.2 to 2.0; *P* = 0.001) may be risk factors for severe adenovirus pneumonia.

**Conclusions:**

Leukocytosis, co-infection with *Mycoplasma pneumoniae*, and high blood viral load may be risk factors for severe adenovirus pneumonia in immunocompetent children. Blood viral load may predict pneumonia severity.

## Background

Early in 1998, a retrospective study of disseminated adenovirus disease in immunocompromised and immunocompetent children found that viremia and prolonged viral shedding were more common in immunocompromised children, although the clinical features and outcomes were similar [1]. Since 2001, quantitative polymerase chain reaction (PCR) has been widely used to detect the adenovirus DNA in the blood of immunocompromised children [2]. Previous studies have demonstrated an association between adenovirus viremia and the risk of both disseminated disease and mortality [3–5]. However, few studies have explored the role of adenovirus viremia in immunocompetent children.

In June 2019, a guideline for the diagnosis and treatment of adenovirus pneumonia in children was published in China in response to an outbreak of human adenovirus infections [6]. An increased number of immunocompetent children with severe adenovirus pneumonia were observed. Possible risk factors for severe adenovirus pneumonia, including viremia, were presumed based on studies of immunocompromised children with adenovirus infections but have not been confirmed in immunocompetent children [3–5]. Little is known about the role of adenovirus viremia in immunocompetent children with adenovirus pneumonia.

In this study, we report the clinical characteristics of adenovirus pneumonia in immunocompetent children and explore the role of adenovirus viremia. We also identify possible risk factors for severe adenovirus pneumonia in immunocompetent children.

## Methods

### Study design

This study was a retrospective, observational study conducted in Shenzhen Children’s Hospital, a 1300-bed tertiary care facility in Shenzhen, China. The study population consisted of all consecutive patients with acute respiratory symptoms hospitalized between May 2019 and August 2019. Children with an immunofluorescence assay or PCR test with respiratory tract specimens that was positive for adenovirus and radiographic findings of pneumonia were included. Children with any of the following factors were excluded: classified in the new-born age group; infection with HIV; leukaemia; known or suspected active tuberculosis; use of immunosuppressive agents; immunodeficiency; chemotherapy; and chronic conditions (malnutrition, congenital heart disease; chronic lung disease).

### Classification of pneumonia severity

The classification of pneumonia severity was based on the criteria in the community-acquired pneumonia guideline in China [7]. Based on the clinical symptoms and chest imaging findings, patients were divided into a severe pneumonia group and a mild pneumonia group. Severe cases were identified on the basis of the presence of at least one of the following signs: disturbance of consciousness, significant tachypnea (respiratory rate > 70 breaths per minute in infants and > 50 breaths per minute in older children), cyanosis, dyspnoea, oxygen saturation < 92%, extrapulmonary complications, dehydration, refusal to eat, and severe chest imaging findings (pneumothorax, pleural effusion, pulmonary atelectasis, or multilobe infiltrates).

### Data collection and management

The clinical variables were measured every day during hospitalization. Blood draws were performed during hospitalization as needed to guide management decisions. Demographic information (age and sex), signs and symptoms (temperature, blood pressure, pulse and respiratory rate, cough, tachypnea, cyanosis, etc.), laboratory results (haematology, organ function, pathogen tests, etc.), chest imaging results including chest x-ray and computed tomography (CT), bronchoscopy results, treatments (oxygen supplementation, endotracheal intubation, antimicrobial agent administration, etc.) and outcome (survival, death, recovery or discharge against medical advice) were recorded. *Mycoplasma pneumoniae* (MP) co-infection was defined as a PCR test of an oropharyngeal swab sample or bronchoalveolar lavage fluid (BALF) that was positive for MP DNA during hospitalization. Viral co-infection was defined as an antigen or PCR test of a nasopharyngeal swab sample or BALF that was positive for other viruses during hospitalization. Bacterial co-infection was defined as positive culture of a single type of bacteria in blood or respiratory tract specimens (sputum, endotracheal aspirate, or BALF) during hospitalization. Fungal co-infection was defined as a positive test for fungi (antigen, antibody, or culture) in blood or respiratory tract specimens with symptoms and chest imaging findings suggestive of fungal infection.

### Sample management and adenovirus detection

All samples were transported to the laboratory within 4 h. Respiratory tract samples were tested for adenovirus with the D3® UltraTM DFA Respiratory Virus Screening and ID Kit (Diagnostic Hybrids, Inc., USA) or the Adenovirus DNA Detection Kit (Shenzhen Puruikang Biotech Co., Ltd). Serum samples were stored at − 80 °C until the adenovirus PCR analysis. Quantification of the adenovirus load in the serum samples was performed with a commercial fluorescence quantitative PCR kit (Daan Gene, Cat. Guangzhou, China) following the protocol provided by the manufacturer. The limit of detection (LOD) was 500 copies/mL.

### Statistical analysis

We performed a univariate correlation analysis of demographic and laboratory variables to determine the statistical significance of the pairwise associations between the severe and mild pneumonia groups. The Mann-Whitney test and chi-square test were used for quantitative and qualitative variables, respectively. We further performed binary logistic regression analysis to identify independent demographic and laboratory risk factors for severe pneumonia.

The log10-transformed concentration of the serum viral load was used as the independent variable in the analysis. Serum viral loads below the LOD were assigned a viral load value of 1 copy/mL (0 log10 copies/mL). Continuous variables are summarized as the means (standard deviation, SD) when they were normally distributed and as the medians (interquartile range, IQR) if they had a skewed distribution. Sex, age, highest white blood cell (WBC) count during hospitalization, MP co-infection, influenza virus co-infection, and highest serum viral load during the disease course were used as independent variables. Data analysis was performed with SPSS 26.0 software. All *P*-values were two-tailed, and *P* <  0.05 was considered to indicate statistical significance.

## Results

Between May 1, 2019, and August 31, 2019, 111 immunocompetent children with adenovirus pneumonia (60 severe, 51 mild) were admitted to the hospital, and all were included (Table 1). The median age was 40 months (IQR 22–64), and 64 patients were male. Bronchoscopy was performed in 47 severe cases and 7 mild cases, where plastic bronchitis was found in 12 severe cases. Five patients were admitted to the intensive care unit (ICU), and two of them underwent endotracheal intubation. None of the patients received anti-adenovirus treatment. All patients were discharged after recovery or improvement.

**Table 1:** Characteristics of Immunocompetent Children with Adenovirus Pneumonia According to Disease Severity

Characteristics	Severe Group (*n* = 60)	Mild Group (*n* = 51)	*P* Value
Demographics			
Male, n (%)	32 (53%)	32 (63%)	0.317
Age, months (IQR)	35 (21–50)	48 (24–72)	0.052
Clinical features, n (%)			
Bronchoscopy	47 (78%)	7 (14%)	–
Plastic bronchitis	12 (20%)	0	–
ICU admission	5 (8%)	0	–
Laboratory tests			
WBC count, 10^9^/L (IQR)	12.86 (8.10–16.77)	10 .30 (7.10–14.14)	0.034
CRP, mg/L (IQR)	33.64 (12.23–68.98)	26.20 (11.70–43.70)	0.091
Co-infection, n (%)	48 (80%)	25 (49%)	0.001
Fungal co-infection, n	2	0	
Bacterial co-infection, n (%)	4 (7%)	4 (8%)	1.000
RSV co-infection, n (%)	4 (7%)	2 (4%)	0.829
Influenza virus co-infection, n (%)	12 (20%)	7 (14%)	0.382
MP co-infection, n (%)	42 (70%)	18 (35%)	< 0.001
Viremia, n (%)	30 (50%)	12 (24%)	0.004
Blood viral load, log10 copies/mL (IQR)	1.385 (0–4.255)	0 (0–0)	0.001

Tests for 7 respiratory viruses (influenza A, influenza B, respiratory syncytial virus, adenovirus, and parainfluenza 1, parainfluenza 2, and parainfluenza 3 viruses), a test for MP, and bacterial cultures were performed in all the patients. Fungal tests were performed in 2 patients whose chest CT findings suggested fungal infections. One patient had a positive BALF culture for Aspergillus, and the other patient had a positive antibody test for Aspergillus. Evidence of a viral, bacterial, or fungal co-infection was found in 73 patients (Table 1). None of the patients had positive blood cultures for bacteria or fungi.

**Fig. 1:**
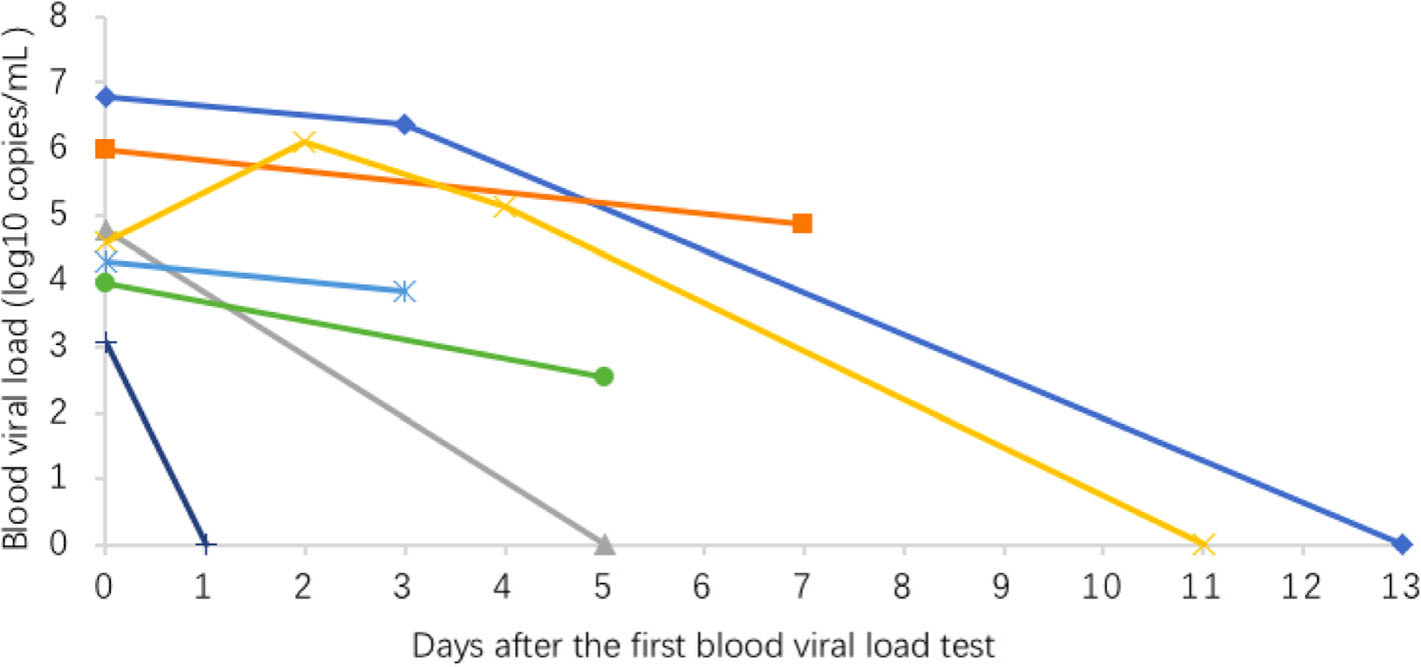
Reduction in adenovirus blood viral load in 7 immunocompetent children with adenovirus pneumonia during recovery. A viral load below the limit of detection was assigned a value of 0 log10 copies/mL

In mild cases, the blood viral load ranged from 0 to 4.54 log10 copies/mL (IQR 0–0). In severe cases, the blood viral load ranged from 0 to 6.78 log10 copies/mL (IQR 0–4.255). The highest blood adenovirus load was observed in a 2-year-old boy with severe pneumonia, plastic bronchitis, pneumothorax, and fungal co-infection. He underwent endotracheal intubation. PCR assays to determine the presence of adenovirus in the blood were performed on the 26th, 29th, and 39th days after disease onset (18th, 21st, and 31st days of hospitalization). The blood viral loads were 6.78 log10 copies/mL, 6.38 log10 copies/mL, and negative, respectively. The reduction in viral load paralleled his clinical recovery, which was also observed in the other 6 patients whose blood viral loads were continuously monitored (Fig. 1). Among the 12 patients with plastic bronchitis, eight developed viremia. Among the five patients admitted to the ICU, three developed viremia. The two patients who underwent endotracheal intubation had the highest and 3rd highest blood viral loads, that is, 6.78 log10 copies/mL and 6.09 log10 copies/mL, of all the blood viral load results in this study.

We identified demographic and laboratory test variables that were significantly associated with severe adenovirus pneumonia. The chi-square test and Mann-Whitney test showed there were significant differences in the WBC count (12.86 × 10^9^/L vs 10.30 × 10^9^/L; *P* = 0.034), co-infection (80% vs 49%; *P* = 0.001), co-infection with MP (70% vs 35%; *P* <  0.001), the presence of viremia (50% vs 24%; *P* = 0.004), and the blood viral load (1.385 log10 copies/mL vs 0 log10 copies/mL; *P* = 0.001) in severe and mild cases.

We performed a binary logistic regression analysis of the following predictors: WBC count, co-infection with MP, and blood viral load (Table 2). In the binary logistic regression analysis, leukocytosis (OR = 1.1; 95% CI: 1.0 to 1.2; *P* = 0.033), co-infection with MP (OR = 5.0; 95% CI: 2.1 to 12.3; *P* <  0.001), and a high blood viral load (OR = 1.5; 95% CI: 1.2 to 2.0; *P* = 0.001) were identified as risk factors for severe adenovirus pneumonia.

**Table 2:** Risk Factors for Severe Adenovirus Pneumonia in Immunocompetent Children

	Severe Group (n = 60)	Mild Group (n = 51)	*P* Value	Odds Ratio (95% CI)
WBC count, 10^9^/L (IQR)	12.86 (8.10–16.77)	10.30 (7.10–14.14)	0.033	1.1 (1.0–1.2)
MP co-infection, n (%)	42 (70%)	18 (35%)	< 0.001	5.0 (2.1–12.3)
Blood viral load, log10 copies/mL (IQR)	1.385 (0–4.255)	0 (0–0)	0.001	1.5 (1.2–2.0)

As co-infection was common among patients in the study and it could affect the predictive value of viremia for disease severity, we also compared the demographics and laboratory test results among the 38 patients without co-infections using Fisher’s exact test and the Mann-Whitney test (Table 3). In the mild pneumonia group, the blood viral load ranged from 0 to 4.54 log10 copies/mL (IQR 0–2.638). In the severe pneumonia group, the blood viral load ranged from 0 to 6.13 log10 copies/mL (IQR 0–4.043). The blood viral load was significantly higher in the severe group.

**Table 3:** Characteristics of Immunocompetent Children with Pneumonia Caused by single Adenovirus Infection

Characteristics	Severe Group (*n* = 12)	Mild Group (*n* = 26)	*P* Value
Demographics			
Male, n (%)	9 (75%)	15 (58%)	0.472
Age (months), IQR	23 (15–42)	41 (22–65)	0.113
Laboratory tests			
WBC count (10^9^/L), IQR	12.51 (6.07–18.10)	10 .40 (6.75–14.51)	0.683
CRP (mg/L), IQR	25.75 (16.50–51.33)	27.45 (19.95–48.33)	0.962
Viremia, n (%)	8 (67%)	11 (42%)	0.295
Blood viral load (log10 copies/mL), IQR	3.675 (0–4.043)	0 (0–2.638)	0.022

## Discussion

From May to August 2019, an outbreak of adenovirus infection occurred in China. Our attention was caught by a common and severe complication of adenovirus infection, namely, adenovirus pneumonia. Because the severity of adenovirus pneumonia in children varies, we explored tools that could be used to predict the severity of this disease and guide management decisions. A previous study of immunocompetent children showed that adenovirus load in respiratory tract secretions was a predictor of the severity of adenovirus pneumonia [8]. Other previous studies in immunocompromised children have demonstrated an association between adenovirus viremia and disease severity [3–5]. It was not clear whether the adenovirus load in the blood could predict adenovirus pneumonia severity in immunocompetent children.

In a study involving 4319 children with respiratory tract infections and 361 controls, 16.4% of the 61 available plasma samples were positive for adenovirus DNA, and they were all from children with infections [9]. There was no comparison made between severe cases and mild cases. In our study, 38% of the patients developed viremia. The blood viral load in severe cases was also significantly higher than that in mild cases, suggesting a positive correlation between the serum viral load and disease severity. Binary logistic regression analysis confirmed the predictive value of the serum viral load for adenovirus pneumonia severity.

In another study of 196 immunocompetent children with adenovirus respiratory tract infections, adenovirus was detected in the blood in 33% of the patients, and there was no difference in ICU admission between the groups with and without viremia [10]. In our study, five patients were admitted to the ICU, and three of them developed viremia. The two patients who underwent endotracheal intubation had the highest and 3rd highest blood viral loads. Although our findings with regard to ICU admission were not comparable with those in the previous study due to the small sample size, they suggested an association between blood viral load and disease severity in patients in the ICU.

In a case series of adenovirus viremia in previously healthy children, a high level of viremia was detected in an adenovirus culture-positive 6-month-old girl with pneumonia, conjunctivitis and hepatitis. The subsequent reduction in viral load paralleled her clinical recovery [11]. Another study of 15 immunocompetent adults with adenovirus pneumonia also showed that the clinical manifestations recovered gradually as the viral load in the blood samples decreased [12]. In our study, seven patients recovered as their blood viral loads decreased, which was consistent with the finding in the previous study.

Our study also identified other possible risk factors for severe adenovirus pneumonia. Previous studies of adenovirus pneumonia suggested that male sex, young age, leukocytosis, and elevated C-reactive protein (CRP) levels were associated with severe pneumonia [13–15]. In our study, male patients were more common in mild cases, which was different from the finding in the previous study. Additionally, although severe cases in our study tended to be younger and have higher CRP levels, there were no significant differences in univariate analyses. Only leukocytosis was significantly associated with severe adenovirus pneumonia, which was consistent with previous studies.

Co-infection with MP has been proposed as a factor involved in the development of severe respiratory infections since 1995 [16]. A recent case-control study in China confirmed that children with MP and adenovirus co-infection had relatively more severe cases than children with MP infections alone [17]. In our study, MP co-infection was common. Logistic regression analysis suggested that it was significantly related to severe adenovirus pneumonia, which was consistent with previous studies.

Co-infection with other pathogens may worsen adenovirus pneumonia and make it difficult to evaluate the predictive value of adenovirus viremia with regard to disease severity. Therefore, we performed a comparison among the 38 patients with pneumonia caused by adenovirus alone. Although the WBC count and number of patients with viremia were not significantly higher in the severe pneumonia group, possibly due to the relatively small sample size, the blood viral load was still significantly higher. This confirmed the predictive value of the blood viral load for the severity of adenovirus pneumonia.

This study has limitations due to its retrospective nature and small sample size. First, a qualitative PCR assay was used to detect adenovirus in respiratory tract specimens during the outbreak. The respiratory tract samples were not saved, making the viral load unclear. Therefore, adenovirus viral loads between respiratory tract samples and blood samples could not be compared. Second, typing of the adenovirus in the blood was not performed due to the insufficiency of the remaining blood samples. Third, an MP PCR assay targeting DNA was used to detect MP, which made it difficult to differentiate patients with active infections from carriers. Detection of MP RNA or paired sera antibody tests would have provided further evidence of active MP infections. Fourth, the sample size of patients without co-infections was small. The statistical analysis would be more accurate if the sample size was larger.

## Conclusions

Leukocytosis, co-infection with *Mycoplasma pneumoniae*, and a high blood viral load may be risk factors for severe adenovirus pneumonia in immunocompetent children. The blood viral load may predict pneumonia severity.

## Data Availability

The datasets used in the current study are available from the corresponding author on reasonable request.

## Abbreviations

BALF: Bronchoalveolar lavage fluid
CI: Confidence interval
CRP: C-reactive protein
CT: computed tomography
ICU: Intensive care unit
IQR: Interquartile range
LOD: Limit of detection
MP: Mycoplasma pneumoniae
OR: Odds ratio
PCR: Polymerase chain reaction
SD: Standard deviation
WBC: White blood cell

## References

[1] Munoz FM, Piedra PA, Demmler GJ (1998). Disseminated adenovirus disease in immunocompromised and immunocompetent children. Clin Infect Dis.

[2] Lankester AC, van Tol MJ, Claas EC, Vossen JM, Kroes AC (2002). Quantification of adenovirus DNA in plasma for management of infection in stem cell graft recipients. Clin Infect Dis.

[3] Lion T, Baumgartinger R, Watzinger F (2003). Molecular monitoring of adenovirus in peripheral blood after allogeneic bone marrow transplantation permits early diagnosis of disseminated disease. Blood..

[4] Echavarria M, Forman M, van Tol MJ, Vossen JM, Charache P, Kroes AC (2001). Prediction of severe disseminated adenovirus infection by serum PCR. Lancet..

[5] Leruez-Ville M, Minard V, Lacaille F (2004). Real-time blood plasma polymerase chain reaction for management of disseminated adenovirus infection. Clin Infect Dis.

[6] National Health Commission of China (2019). Guideline for diagnosis and treatment of adenovirus pneumonia in children (2019 version). Chin J Clin Infect Dis.

[7] National Health Commission of China (2019). Guideline for diagnosis and treatment of community-acquired pneumonia in Children (2019 version). Chin J Clin Infect Dis.

[8] Xie L, Zhang B, Zhou J (2018). Human adenovirus load in respiratory tract secretions are predictors for disease severity in children with human adenovirus pneumonia. Virol J.

[9] Schjelderup NH, Nordbo SA, Krokstad S, Dollner H, Christensen A (2019). Human adenovirus in nasopharyngeal and blood samples from children with and without respiratory tract infections. J Clin Virol.

[10] Song E, Wang H, Leber A, Jaggi P (2017). Human adenovirus (HAdV) Viremia in Immunocompetent children with HAdV infection in respiratory specimens: does Viremia predict severity of illness?. Open Forum Infect Dis.

[11] Shike H, Shimizu C, Kanegaye J, Foley JL, Burns JC (2005). Quantitation of adenovirus genome during acute infection in normal children. Pediatr Infect Dis J.

[12] Gu L, Qu J, Sun B, Yu X, Li H, Cao B (2016). Sustained Viremia and high viral load in respiratory tract secretions are predictors for death in Immunocompetent adults with adenovirus pneumonia. PLoS One.

[13] Chuang Y, Chiu CH, Wong KS (2003). Severe adenovirus infection in children. J Microbiol Immunol Infect.

[14] Miao H, Rong L, Zhou F (2017). Risk factors for poor prognosis in children with severe adenovirus pneumonia. Chin J Contemp Pediatr.

[15] Mingyue L, Linying G, Dong Q (2018). Clinical analysis of children in hospital with adenovirus pneumonia in Beijing from 2015 to 2016. Chin J Exp Clin Virol.

[16] Cimolai N, Wensley D, Seear M, Thomas ET (1995). Mycoplasma pneumoniae as a cofactor in severe respiratory infections. Clin Infect Dis.

[17] Gao J, Xu L, Xu B, Xie Z, Shen K (2020). Human adenovirus Coinfection aggravates the severity of mycoplasma pneumoniae pneumonia in children. BMC Infect Dis.

